# Novel *MT-ND* Gene Variants Causing Adult-Onset Mitochondrial Disease and Isolated Complex I Deficiency

**DOI:** 10.3389/fgene.2020.00024

**Published:** 2020-02-25

**Authors:** Yi Shiau Ng, Kyle Thompson, Daniela Loher, Sila Hopton, Gavin Falkous, Steven A. Hardy, Andrew M. Schaefer, Sandip Shaunak, Mark E. Roberts, James B. Lilleker, Robert W. Taylor

**Affiliations:** ^1^ Wellcome Centre for Mitochondrial Research, Faculty of Medical Sciences, Translational and Clinical Research Institute, Newcastle University, Newcastle upon Tyne, United Kingdom; ^2^ Directorate of Neurosciences, Royal Victoria Infirmary, The Newcastle upon Tyne Hospitals NHS Foundation Trust, Newcastle upon Tyne, United Kingdom; ^3^ Faculty of Medicine, Institute of Biochemistry and Molecular Biology, ZBMZ, University of Freiburg, Freiburg, Germany; ^4^ Newcastle upon Tyne Hospitals NHS Foundation Trust, NHS Highly Specialised Service for Rare Mitochondrial Disorders, Newcastle upon Tyne, United Kingdom; ^5^ Department of Neurology, Royal Preston Hospital, Preston, United Kingdom; ^6^ Manchester Centre for Clinical Neuroscience, Salford Royal NHS Foundation Trust, Manchester Academic Health Science Centre, Salford, United Kingdom; ^7^ Centre for Musculoskeletal Research, Division of Musculoskeletal and Dermatological Sciences, School of Biological Sciences, The University of Manchester, Manchester, United Kingdom

**Keywords:** mitochondrial DNA, muscle biopsy, myopathy, deafness, tissue segregation

## Abstract

Mitochondrial complex I deficiency is associated with a diverse range of clinical phenotypes and can arise due to either mitochondrial DNA (mtDNA) or nuclear gene defects. We investigated two adult patients who exhibited non-syndromic neurological features and evidence of isolated mitochondrial complex I deficiency in skeletal muscle biopsies. The first presented with indolent myopathy, progressive since age 17, while the second developed deafness around age 20 and other relapsing-remitting neurological symptoms since. A novel, likely *de novo,* frameshift variant in *MT-ND6* (m.14512_14513del) and a novel maternally-inherited transversion mutation in *MT-ND1* were identified, respectively. Skewed tissue segregation of mutant heteroplasmy level was observed; the mutant heteroplasmy levels of both variants were greater than 70% in muscle homogenate, however, in blood the *MT-ND6* variant was undetectable while the mutant heteroplasmy level of the *MT-ND1* variant was low (12%). Assessment of complex I assembly by Blue-Native PAGE demonstrated a decrease in fully assembled complex I in the muscle of both cases. SDS-PAGE and immunoblotting showed decreased levels of mtDNA-encoded ND1 and several nuclear encoded complex I subunits in both cases, consistent with functional pathogenic consequences of the identified variants. Pathogenicity of the m.14512_14513del was further corroborated by single-fiber segregation studies.

## Introduction

Mitochondrial NADH:ubiquinone oxidoreductase (Complex I) is the first and largest (~1 MDa) complex of the mitochondrial respiratory chain involved in the oxidative phosphorylation (OXPHOS) pathway and generation of ATP. It comprises 45 structural subunits of which seven are encoded by mitochondrial DNA (mtDNA), the remaining subunits being encoded by the nuclear genome as are the ~20 ancillary proteins required for assembly and biogenesis (Formosa et al., 2018). As such, genetic defects in both mitochondrial and nuclear DNA can result in isolated complex I deficiency.

Complex I deficiency is the most common biochemical defect associated with mitochondrial disease (Alston et al., 2017). Identical biochemical defects are associated with phenotypic heterogeneity, (Kirby et al., 1999; Janssen et al., 2006) ranging from a tissue specific manifestations such as Leber hereditary optic neuropathy (LHON), (Man et al., 2002) to devastating, severe phenotypes including Leigh syndrome, (Distelmaier et al., 2009) mitochondrial encephalomyopathy, lactic acidosis and stroke-like episodes (MELAS) syndrome, multi-system disease, (Alston et al., 2010) and hypetrophic cardiomyopathy and severe lactic acidosis (Distelmaier et al., 2009). Pathogenic variants have been identified in all seven mtDNA-encoded subunits of complex I; however, there is no clear genotype-phenotype correlation (Distelmaier et al., 2009; Hoefs et al., 2012). While incomplete penetrance is frequently observed in the homoplasmic variants associated with LHON, (Man et al., 2002) the heteroplasmy levels in other pathogenic variants such as m.13513G > A and m.13094T > C in *MT-ND5,* both reported in Leigh Syndrome and MELAS syndrome, show good correlation with the severity of disease burden (Ng et al., 2018). Conversely, some *de novo* pathogenic variants in the *MT-ND* (Mitochondrially-encoded NADH:ubiquinone oxidoreductase core subunit) genes cause slowly progressive, non-syndromic presentations such as myopathy and exercise intolerance (Gorman et al., 2015).

In this report, we present two adult patients with complex I deficiency manifesting with different clinical pictures, one developing an insidious-onset myopathy while the other presents with deafness in her 20s and subsequent neurological symptoms that follow a relapsing-remitting pattern. Novel variants in the mtDNA-encoded MT-ND6 and MT-ND1 proteins were identified, respectively, and characterized fully to demonstrate causality.

## Material and Methods

### Case Reports

#### Patient 1

A 27-year-old man was referred to a neurology service with a 10-year history of exercise intolerance and mild muscle weakness. In addition, the patient also complained of intermittent drooping of his eyelids and double vision. There was no history of myoglobinuria, deafness, optic atrophy, or retinitis pigmentosa. There was no family history of neuromuscular disorder. Clinical examination revealed very mild proximal lower limb weakness with MRC grade 4+/5. The upper limb muscle bulk was reduced, and subtle scapular winging, and an excessive lumbar lordosis were apparent. The rest of the neurological examination was normal. Routine laboratory investigations were normal except for an elevated serum creatine kinase (CK) (1,212 IU/L). He underwent electromyography (EMG) study which showed polyphasic myopathic units in most muscles sampled. No myotonia or abnormal decrement was evident. He was found to have dipstick proteinuria, and a 24-h urine collection confirmed the presence of microalbuminuria. The following investigations were either negative or normal: serum lactate level, anti-acetylcholine receptor and anti-muscle specific kinase autoantibodies, forearm ischaemic lactate test, serum alpha-glucosidase levels, cardiac investigations (including ambulatory electrocardiogram (ECG) and echocardiogram), renal ultrasound scan, magnetic resonance imaging (MRI) of the brain, and MRI of the upper and lower limb muscles. He had a muscle biopsy at the age of 28 years.

#### Patient 2

This patient presented with painless, sequential visual loss over four months during pregnancy at the age of 35 years. Her visual acuity at the nadir was documented to be 6/60 bilaterally with the presence of relative afferent pupillary defect in one eye. Her medical history included endometriosis, gestational diabetes and hearing impairment since her late 20s. Both her mother and maternal grandmother developed hearing impairment in their 40s. Retrobulbar optic neuritis was initially suspected, however, her MRI head (including angiography) did not identify any acute structural changes and the visual evoked potentials (VEP) were normal. Her vision gradually improved over several months. Three years later, she developed a gradual-onset, severe headache. MRI head showed several subcortical T2 hyperintensities. The possibility of raised intracranial pressure was excluded with normal cerebrospinal fluid (CSF) opening pressure. CSF constituents were normal, and CSF-restricted oligoclonal bands were not detected. Her headache settled a week later. At age 44 years, she presented with left arm weakness. A CT head scan was normal and the weakness improved spontaneously a week later. Three months later, she re-presented with vertigo, poor balance, sensory disturbances on the left hand, and fatigue. A repeat MRI head showed an increase in subcortical and periventricular white matter lesions with sparing of the corpus callosum. However, repeat CSF studies and VEP remained unremarkable. Her resting serum lactate was 1.2 mmol/L (normal < 2.2 mmol/L). At this point, mitochondrial disease was considered, and a muscle biopsy was performed. In the last clinical review at the age of 46, she developed diabetes mellitus and complained of unsteadiness and fatigue. She had a mild dysarthric speech and reduced muscle strength in the hip flexion (MRC grade 4+/5). Her recent cardiac investigations were normal.

### Histochemical and Quadruple Immunohistochemistry (IHC) Studies of Diagnostic Muscle Biopsies

Standard histological (modified Gomori trichrome) and histochemical (individual cytochrome c oxidase (COX), succinate dehydrogenase (SDH), and sequential COX-SDH) analyses of skeletal muscle biopsies were performed on fresh-frozen skeletal muscle sections (10 µm) as previously described (Old and Johnson, 1989). Quadruple OXPHOS immunofluorescence was undertaken on transversely-orientated frozen muscle sections (10 µm) according to a previously validated protocol to establish evidence of complex I or complex IV deficiency (Rocha et al., 2015).

### Molecular Genetic Analyses

Total DNA was extracted from available tissues including sketelal muscle, blood, buccal epithelia, and urinary sediments. In both patients, muscle mtDNA rearrangements were investigated using several long-range PCR strategies prior to sequencing of the entire mitochondrial genome as described elsewhere (Krishnan et al., 2007; Zierz et al., 2019). Analytical sensitivity for single nucleotide variants present at ≥5% heteroplasmy is ≥95% (95% confidence intervals).

### Assessment of mtDNA Mutation Load by Quantitative Pyrosequencing

Mutation loads of m.14512_14513del *MT-ND6* and m.3761C > A *MT-ND1* variants were determined in homogenate tissue by quantitative pyrosequencing; quantification of the heteroplasmy level of each variant was achieved using Pyromark Q24 software (Grady et al., 2018). For Patient 1 (m.14512_14513del mutation), we also determined the mutation loads in individual, laser-microdissected muscle fibers for two groups: COX-positive reacting fibers and COX-positive, ragged-red fibers showing marked subsarcolemmal mitochondrial accumulation.

### BN–PAGE and Western Blot Analysis of Patient Muscle

Blue-Native Polyacrylamide Gel Electrophoresis (BN**–**PAGE) was performed using mitochondrial proteins isolated from skeletal muscle samples (25 mg of tissue) as described previously (Thompson et al., 2016) using antibodies against COXI (abcam ab14705), SDHA (abcam ab14715), VDAC1 (abcam ab14734), UQCRC2 (abcam ab14745), NDUFB8 (abcam ab110242), and ATP5A (abcam ab14748); all primary antibodies were used at a dilution of 1 in 1,000. Total protein extraction from human muscle for sodium dodecyl sulphate-polyacrylamide gel electrophoresis (SDS-PAGE) and western blotting was carried out as described (Olahova et al., 2015) using the following commercially available antibodies: NDUFB8 (abcam ab110242), NDUFV1 (Proteintech 11238-1-AP), NDUFS3 (abcam ab110246), SDHA (abcam ab14715), UQCRC2 (abcam ab14745), COXI (abcam ab14705), ATP5A (abcam ab14748), and VDAC1 (abcam ab14734), which served as a loading control. The antibody against ND1 was a kind gift from Dr Anne Lombès.

## Results

### Histochemical and Quadruple Immunohistochemistry (IHC) Studies of Muscle Biopsy

**Figure 1:**
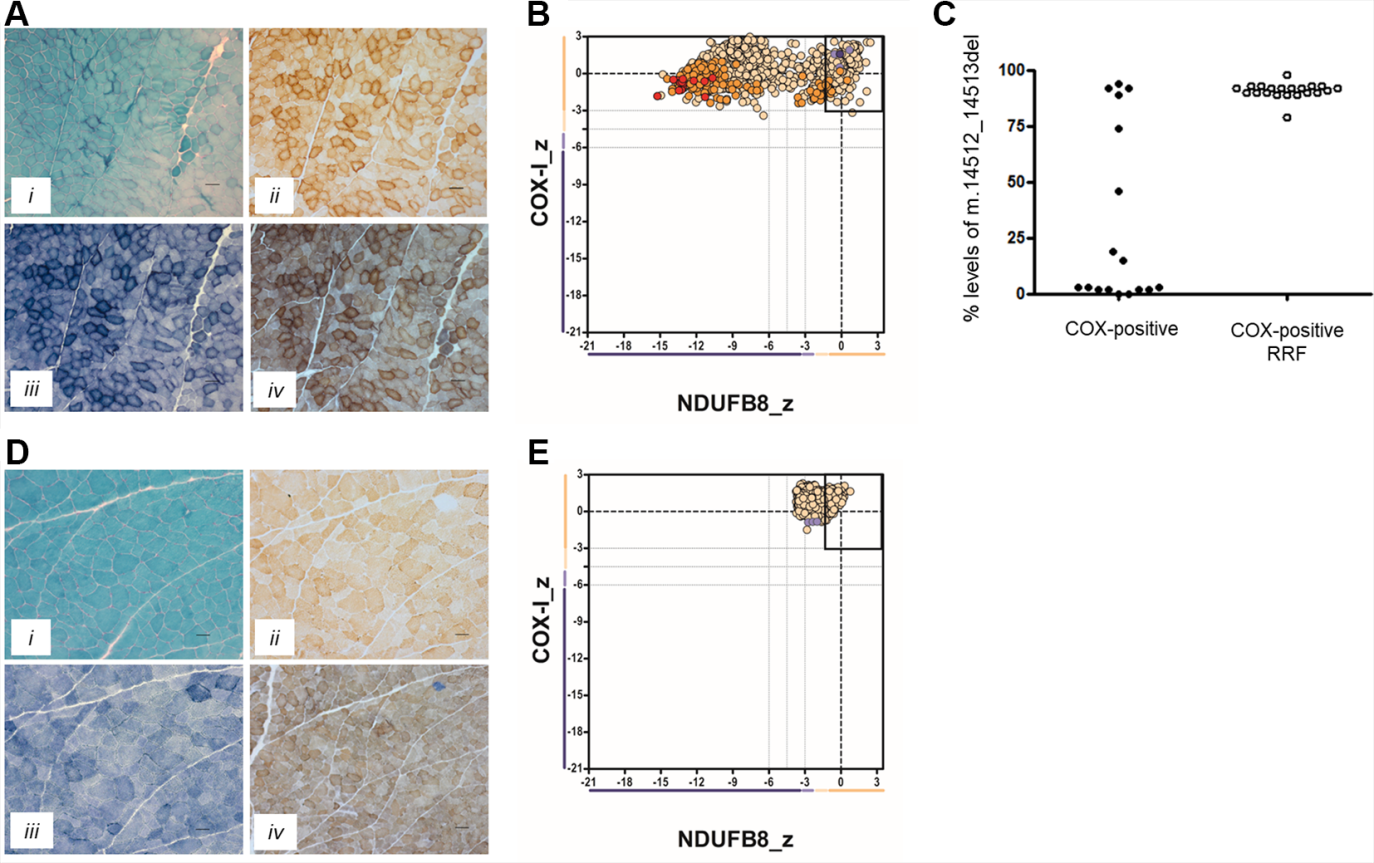
Muscle biopsy findings in two patients with isolated complex I deficiency. **(A)** Histopathological analysis of skeletal muscle sections from Patient 1 showing modified Gomori trichrome staining (i), cytochrome *c* oxidase (COX) histochemistry (ii), succinate dehydrogenase (SDH) histochemistry (iii), and sequential COX-SDH histochemistry (iv), highlighting the presence of COX-positive ragged-red fibers (RRF) showing mitochondrial accumulation. Scale bars = 100 µm. **(B)** Respiratory chain profile following quadruple oxidative phosphorylation immunofluorescence analysis of cryosectioned muscle from Patient 1, confirming the presence of numerous fibers lacking complex I (NDUFB8) protein. Each dot represents the measurement from an individual muscle fiber, color coded according to its mitochondrial mass (blue-low, normal-beige, high-orange, very high-red). Gray dashed lines indicate SD limits for the classification of fibers. Lines next to x- and y-axes represent the levels (SDs from the average of control fibers after normalization to porin/VDAC1 levels; _z = Z-score, see Methods section of Rocha et al. (2015) for full description of statistics (Rocha et al., 2015) of NDUFB8 and COX1, respectively (beige = normal (>−3), light beige = intermediate positive (−3 to −4.5), light purple = intermediate negative (−4.5 to −6), purple = deficient (<−6). Bold dotted lines indicate the mean expression level observed in respiratory normal fibers. **(C)** Single fiber PCR analysis shows significant segregation of higher m.14512_14513del, p.(Met54Serfs*7) *MTND6* mutation load within COX-positive RRF than COX-positive fibers not showing obvious subsarcolemmal mitochondrial accumulation. **(D)** Histopathological analysis of skeletal muscle sections from Patient 2 showing modified Gomori trichrome staining (i), COX histochemistry (ii), SDH histochemistry (iii), and sequential COX-SDH histochemistry (iv). COX-SDH histochemistry identified a single, COX-deficient fiber which is likely the result of somatic (age-related) mtDNA mutation. **(E)** Respiratory chain profile following quadruple oxidative phosphorylation immunofluorescence analysis of cryosectioned muscle from Patient 2, again confirming the presence of fibers lacking complex I (NDUFB8) protein.

In Patient 1, the oxidative enzyme reactions (SDH and COX) revealed numerous fibers with increased activity at the fiber periphery, confirmed by modified Gomori trichrome staining, which showed subsarcolemmal accumulations typical of “ragged-red” changes affecting >30% of all fibers (**Figure 1A**). Quadruple OXPHOS IHC assay detected >75% of fibers showing a complete loss of NDUFB8 immunoreactivity, again associated with preserved COX-I immunoreactivity (**Figure 1B**). Many of these fibers showed high porin levels, reflecting enhanced mitochondrial numbers fibers showing subsarcolemmal mitochondrial accumulation. A histopathological assessment of the muscle biopsy from Patient 2 failed to detect significant mitochondrial changes; a single COX-deficient fiber was noted following sequential COX-SDH histochemistry, likely as a result of somatic mtDNA mutation (**Figure 1D**). However, the IHC mitochondrial respiratory chain profile shows a loss of NDUFB8 immunoreactivity, associated with preserved COX-I immunoreactivity, for >60% of all fibers and consistent with isolated complex I deficiency (**Figure 1E**).

### Identification of Novel Pathogenic *MT-ND6* and *MT-ND1* Mutations

Long-range PCR assays were used to exclude mtDNA rearrangements in the muscle from both patients, prompting the sequencing of the entire mitochondrial genome which identified candidate pathogenic variants in genes encoding structural subunits of mitochondrial complex I. We determined the mtDNA sequence in muscle from both patients identifying novel, candidate pathogenic *MTND* mutations. Patient 1 was shown to harbor a novel m.14512_14513del, p.(Met54Serfs*7) variant, also predicting the premature truncation of the relevant complex I protein subunit (ND6). Quantitative pyrosequencing showed that the m.14512_14513del variant was present at high levels of heteroplasmy in skeletal muscle (76%); at low levels (10%) in a urinary sediment-derived DNA sample but undetectable in blood and buccal epithelial-derived DNA samples. Concurrent studies in his mother’s blood, urine and buccal epithelial DNA samples failed to detect the m.14512_14513del variant, strongly implicating a *de novo* mutation event.

Patient 2 harbored a novel m.3761C > A transversion (predicting p.(Ser152*) and the premature truncation of the ND1 protein) which was present at high levels of heteroplasmy in skeletal muscle (80%), and lower levels in other tissues including urinary sediment (46%), buccal epithelia (35%), and blood (12%). Testing of the samples from the patients clinically-unaffected mother confirmed maternal transmission of the m.3761C > A variant, with lower levels of mtDNA heteroplasmy detected in urinary sediments (38%) and blood (5%).

Neither the m.14512_14513del nor m.3761C > A variants were reported within online databases of mtDNA variation, nor did we detect these within our own in-house database of >1,950 human mtDNA sequences. Using quantitative pyrosequencing, we detected significantly higher levels of the m.14512_14513del variant in COX-positive ragged-red fibers [90.9 ± 0.74% (*n* = 21)] than in COX-positive non-ragged-red fibers [31.7 ± 9.6% (*n* = 17)] (p < 0.0001, two-tailed Student’s *t* test), confirming segregation of the m.14512_14513del genotype with a histopathological abnormality in Patient 1 (**Figure 1C**).

### Novel *MTND* Gene Mutations are Associated With Impaired Complex I Assembly and Loss of Immunoreactive Complex I Subunits

**Figure 2:**
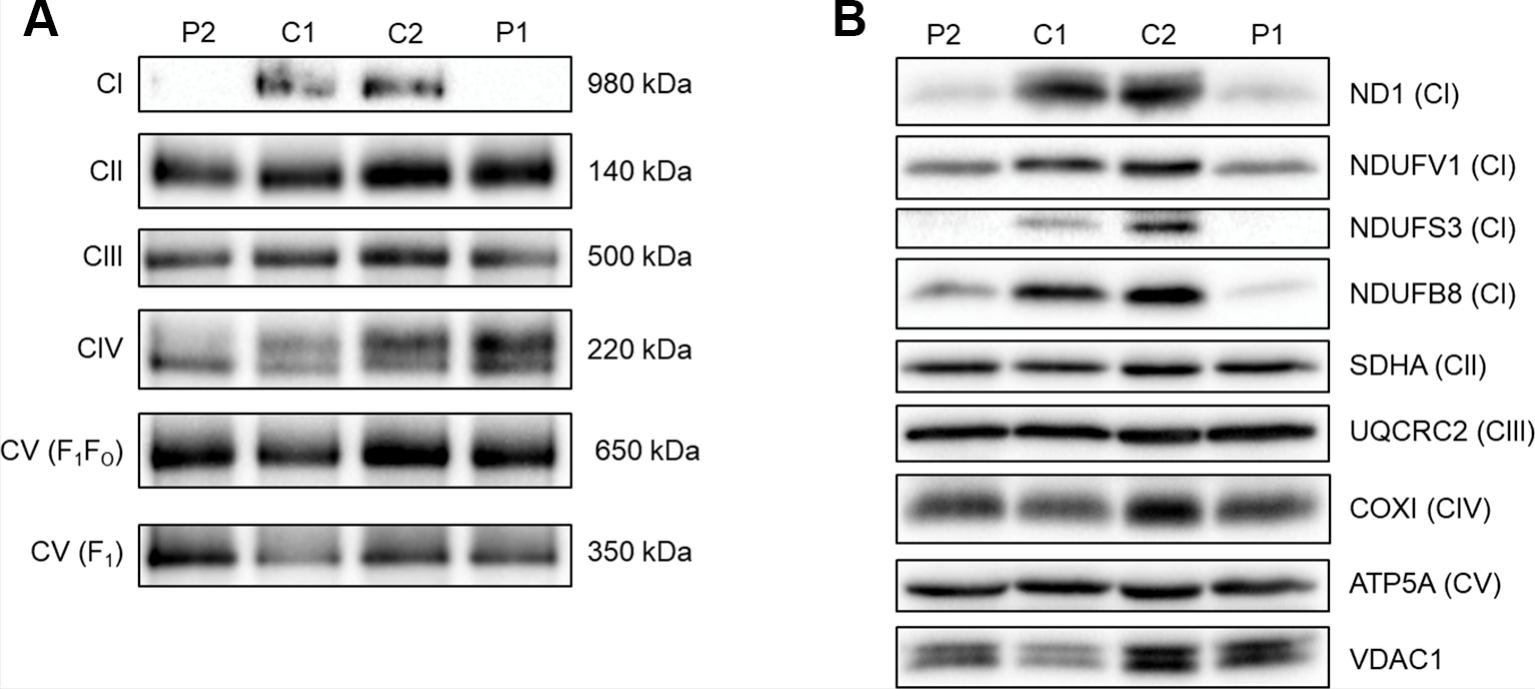
Assessing OXPHOS complex assembly and protein levels in patient muscle. **(A)** BN-PAGE of muscle samples from two age-matched controls (C1 and C2) and Patients (P1 and P2). Antibodies used were anti-NDUFB8 for complex I (CI), anti-SDHA for complex II (CII), anti-UQCRC2 for complex III (CIII), anti-COX1 for complex IV (CIV), and anti-ATP5A for complex V (CV). Complex II was used as a loading control. Blots are representative of two technical repeats. **(B)** SDS-PAGE and immunoblotting analysis of muscle samples from two age-matched controls (C1 and C2) and Patients (P1 and P2). Antibodies against ND1, NDUFV1, NDUFS3, NDUFB8 were used as markers of complex I; SDHA for complex II; UQCRC2 for complex III; COXI for complex IV; ATP5A for complex V and VDAC1 as a mitochondrial mass marker. Blots are representative of two independent experiments.

To assess the ability of complex I to assemble in the inner mitochondrial membrane, a one-dimensional BN-PAGE was performed with muscle samples isolated from both patients and two age-matched healthy controls. A band representing fully assembled complex I (980 kDa) was detectable in both controls, but Patient 2 showed very weak signal and no signal was detected in Patient 1 (**Figure 2A**). However, the assembly of all other OXPHOS complexes were unchanged between patients and controls, confirming an isolated complex I defect in skeletal muscle from both patients. SDS-PAGE and immunoblotting was performed in skeletal muscle samples from each patient and showed a decrease in the steady-state protein levels of all complex I subunits tested (ND1, NDUFV1, NDUFS3, and NDUFB8) (**Figure 2B**), whereas subunits of complexes II-V (SDHA, UQCRC2, COXI, and ATP5A, respectively) were unchanged between patients and controls.

## Discussion

Mitochondrial disease presentations that do not exhibit classical syndromic clinical phenotypes can be difficult to diagnose. Both patients described in this report have undergone multiple investigations over several years, with the eventual diagnosis being underpinned by clear evidence of mitochondrial complex I deficiency in a diagnostic muscle biopsy.

A heteroplasmic m.14512_14513del *MT-ND6* variant was identified in Patient 1 who presented with exercise intolerance, mild myopathy, and hyperCKaemia. This novel mtDNA variant has likely arisen *de novo* as it is not detectable in several mitotic tissues of his clinically-unaffected mother although we demonstrate skewed tissue segregation of this variant in the patient. A novel, m.3761C > A; p.(Ser152*) *MT-ND1* variant is the likely cause of Patient 2’s personal and maternal history of deafness and her relapsing-remitting neurological presentations. Both mtDNA variants clearly result in the isolated complex I deficiency, as identified with the IHC findings of decreased expression of complex I subunit (NDUFB8), BN-PAGE showing perturbed assembly of the complex I holoenzyme and immunobloting showing a decrease in the steady-state protein of complex I subunits. Moreover, the pathogenicity of the m.14512_14513del variant is further supported by the single-fiber segregation analysis confirming higher levels of the variant are present in ragged-red fibers.

The maternal inheritance of the m.3761C > A in *MT-ND1* variant and the observed decrease in ND1 protein levels in skeletal muscle samples from Patient 2 strongly indicate pathogenicity of this variant. Skeletal muscle from Patient 1, harboring the m.14512_14513del variant in *MT-ND6,* also had decreased ND1 protein levels as well as decreased levels of several nuclear encoded complex I subunits (NDUFV1, NDUFS3, and NDUFB8). This is consistent with decreased ND6 levels leading to a complex I assembly defect and subsequent degradation of many complex I subunits and is similar to what is seen in Patient 2 due to the loss of ND1. ND6 could not be directly assessed by immunoblotting due to the lack of availability of an antibody to ND6. In both patients, NDUFV1 is the least affected subunit. This is likely due to NDUFV1 being part of the N module of complex I which is assembled separately to the Q/ND1 and ND2 modules that ND1 and ND6 are part of respectively (Mimaki et al., 2012; Formosa et al., 2018).

Progressive exercise intolerance and myopathy identified in Patient 1 are infrequent clinical findings associated with pathogenic *MT-ND* variants (Musumeci et al., 2000; Gorman et al., 2015). The putative link between the mitochondrial complex I defect and glomerular dysfunction is highly conceivable given no other cause has been identified, and renal involvement is increasingly recognized as part of the multisystem manifestation in mitochondrial diseases (O’Toole, 2014).

A retrospective review of the history of bilateral visual impairment in Patient 2 raised the suspicion of LHON. However, the details of initial retinal examination were not available and it is not known whether characteristic acute findings of LHON such as disc hyperemia, oedema of the peripapillary retinal nerve fiber layer andretinal telangiectasia were evident . The relapsing-remitting nature of subsequent neurological presentations mimicked multiple sclerosis but the radiological, VEP and CSF findings were not supportive of the diagnosis. While there are some uncertainties on establishing the causal link between visual disturbance, white matter changes and the novel *MT-ND1* variant, the presence of sensorineural hearing loss, the development of diabetes mellitus, myopathy and maternal history of deafness are typical findings in primary mtDNA disease.

Next generation sequencing (NGS) technology has been increasingly integrated in the diagnostic pathway of a wide range of genetic disorders including mitochondrial disease (Thompson et al., 2019). One of the proposed advantages is that NGS could mitigate the need and the risk of invasive, diagnostic muscle biopsies, especially in the paediatric population. However, primary mtDNA mutations account for two-third of the diagnosis of adult cases, (Gorman et al., 2015) and the skewed segregation of some mtDNA mutations between non-invasive tissues (e.g., blood) and post-mitotic tissues (e.g., muscle) could pose a significant challenge on the interpretation of any variant of unknown significance detected at low heteroplasmy levels in blood-derived DNA. Moreover, the expression of some mtDNA mutations is tissue specific and testing the blood-derived DNA alone could yield a false negative finding, such as in Patient 1 and other reported cases (Andreu et al., 1999; Musumeci et al., 2000). Given these diagnostic caveats listed above, muscle biopsy would retain its crucial role in establishing the diagnosis of primary mtDNA disease, (Hardy et al., 2016; Zierz et al., 2019) especially in cases without apparent maternal history and *de novo* variants.

In conclusion, isolated complex I deficiency is associated with an increasingly diverse phenotypic expression of mitochondrial disease. We highlight two novel mutations causing isolated complex I deficiency and diverse clinical features. Our findings also serve to highlight the importance of diagnostic muscle biopsy in proving the pathogenicity of novel mtDNA variants, particularly in cases with non-syndromic presentations.

## Data Availability Statement

Both novel mtDNA variants have been submitted to ClinVar (https://www.ncbi.nlm.nih.gov/clinvar/), with the following Accession Numbers: m.14512_14513del (SCV001132040); m.3761C>A (SCV001132041).

## Ethics Statement

Ethical review and approval was not required for the study on human participants in accordance with the local legislation and institutional requirements. The patients/participants provided their written informed consent to participate in this study. Written informed consent was obtained from the individual(s) for the publication of any potentially identifiable images or data included in this article.

## Author Contributions

RT has full access to all the data in the study and takes responsibility for the integrity of the data and the accuracy of the data analysis. Study concept and design: YN, KT, and RT. Acquisition, analysis, or interpretation of data: all authors. Drafting of the manuscript: YN, KT, JL and RT. Critical revision of the manuscript for important intellectual content: all authors.

## Conflict of Interest

The authors declare that the research was conducted in the absence of any commercial or financial relationships that could be construed as a potential conflict of interest.
